# Gene-drive mosquitoes: a prospect for future malaria control

**DOI:** 10.11604/pamj.2022.41.109.31687

**Published:** 2022-02-08

**Authors:** Syed Abdullah Monawwer, Ayham Omar Ibrahim Alzubaidi, Farah Yasmin, Shahed Mohammad Qasem Haimour, Syed Muhammad Ismail Shah, Irfan Ullah

**Affiliations:** 1 Department of Internal Medicine, Ziauddin Medical University, Karachi, Sindh, Pakistan,; 2 Jordan University Hospital, Amman, Jordan,; 3 Department of Internal Medicine, Dow Medical College, Dow University of Health Sciences, Karachi, Pakistan,; 4 Kabir Medical College, Gandhara University, Peshawar, Pakistan

**Keywords:** Malaria, malaria control, gene-drive

## Abstract

Despite major developments in malaria control over the past two decades, the disease continues to scourge the human population across the globe. Rising concerns such as insecticide resistance amongst vector mosquitoes are a cause of huge fear amongst healthcare providers and policymakers. Amidst such dire circumstances, a recent development may form the blueprint for future malaria control as for the first time ever researchers were able to decimate an entire mosquito population using gene-drive technology within a span of one year in a multi-generation, ecologically challenging study. Despite some concerns, the technology displayed a high potential of becoming a powerful tool in malaria control.

## Commentary

Despite continuous efforts to curtail and eradicate malaria, it remains one of the most daunting scourges of public health of the twenty-first century [1]. Although preventable and curable, the disease continues to cause high morbidity and mortality [2]. According to the World Health Organization´s malaria report of 2020, in 2019, almost half the world´s population was at risk of contracting the disease. There were an estimated 220 million cases of Malaria globally, causing an approximate of 409,000 deaths the same year [2]. A staggering 94% of these cases originated in Africa alone in addition to the eastern Mediterranean, Southeast Asian, American, and Western Pacific regions [2].

Malaria is caused by the plasmodium parasite and to-date, five species of plasmodium are known to infect humans; *P. ovale, P. knowlesi, P. malariae, P. vivax* and *P. falciparum*. Of these, *P. falciparum* and *P. vivax* are known to be the most lethal with 90% of all cases and almost all deaths being attributed to *P. falciparum* [1]. The parasite is transmitted by its vector, the Anopheles mosquitoes. As of now, 70 different species of the Anopheles are known to be competent vectors for malaria with majority of malarial cases in African countries being attributed to the *Anopheles funestus*, and three species of *Anopheles gambiae* [1].

The pathophysiological mechanisms underlying the disease involves the release of inflammatory cytokines induced by the malarial toxin. Patients infected with malaria experience symptoms including typical fever cycles, nausea, vomiting, chills and headache within two weeks of the mosquito bite [1,2]. With immediate medical attention these symptoms can easily resolve, however if not timely treated, these can progress to serious life-threatening complications such as severe anemia, cerebral malaria, coma or even death [3]. It must however be noted that often in *P. Vivax* and *P. Ovale* infections, the parasite may display dormant schizogony where it remains dormant for a period of months to years until it is reactivated. Groups most vulnerable to severe malarial complications comprise pregnant women, children under five years old, immunocompromised, and travelers to endemic areas [1]. In 2019 children under five accounted for 67% of all malaria related deaths worldwide. Apart from causing high morbidity and mortality, the disease has been a huge deterrent to social and economic stability from individual to government level. Afflicting some of the most impoverished regions of the world, the disease thwarts economic activities and gives rise to huge treatment costs imposing a huge burden on the people as well as the government making it of the utmost importance to control the spread and work towards the elimination of malaria [2,4].

Malaria prevention and control is centered around chemoprophylaxis, and vector control strategies using insecticide treated nets (ITNs) which provide both a physical barrier and an insecticidal effect against malaria. Another powerful mode to utilize insecticides is through indoor residual spraying (IRS) which offers strong community protection. The WHO also recommends administering prophylactic antimalarial drugs to all individuals at risk. Further travelers from non-endemic areas are counseled regarding preventative measures and given prophylactics [2,4]. Currently, the first and only anti-malarial vaccine, RTS,S/AS01 (RTS,S) is in its trial phase. The vaccine acts against the deadly *P. falciparum* and through four doses it may play a potential role in protecting young African children from morbidity and death. Several other methods have also been employed to curtail malarial spread including swarm sprays, spatial repellents, mass drug administration, house improvement through installations of screens and closure of eaves, and finally larval source management through biological control, habitat modification, and manipulation [1].

As of 2019, an estimated US $3 billion had been spent on malaria control, and elimination programs which led to increased intervention coverage, and an overall upscale in the struggles against malaria [1,2]. Although these findings are encouraging, several hurdles may greatly hamper, and regress the gains made over the past 20 years. It has been reported that since 2016, ITN coverage has been impeded to a standstill. Similar findings were observed with IRS protection which saw a decline in usage from 5% in 2010 to 2% in 2019 [2]. This is mainly due to affordability issues as even though these measures are considered cheap around the globe, they pose a huge financial burden on the vulnerable population who mainly belong to the lower class. Further, there has been a huge gap between ownership and utilization of the ITN as even though several governments and non-government organizations have been providing the nets free of cost, they are used incorrectly or inconsistently [1]. Moreover, 73 countries have reported mosquito resistance to at least one of the four commonly used insecticides. Likewise, resistance to antimalarial drugs has been a recurrent problem, halting the progress made in improving childhood survival [2]. Another factor that may greatly increase the malaria burden is the rising average global temperature and the changing weather patterns that may favor mosquito survival and facilitate transmission [3,4]. It is hypothesized that a 3°C rise in temperature can increase the incidence of malaria by 50 to 80 million [3]. All such adverse findings may jeopardize the current momentum of malaria reduction and elimination, and call for more robust and sustainable tools in the global response to malaria [1].

**Figure 1:**
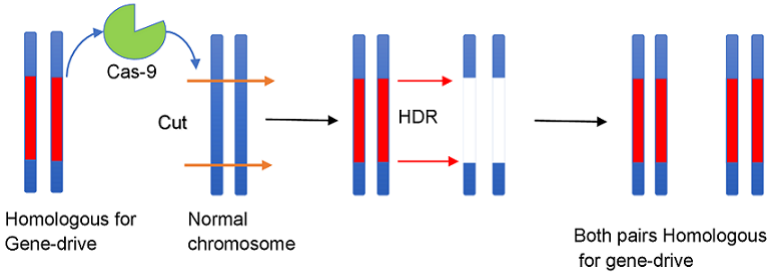
process of homing

**Figure 2:**
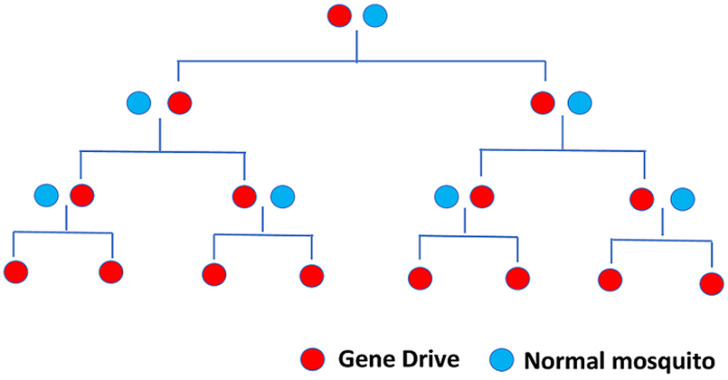
inheritance pattern of gene-drive

Amidst these rising concerns, a very recently made development forms what may be the blueprint for future malaria control. Gene-drive technology has been proven to successfully decimate an *Anopheles Gambiae* mosquito population within a span of one year in an overlapping generation study conducted in large cages that reproduced complex behaviors and challenges found in mosquitoes in the open. This makes it the first instance where the efficacy for a gene-drive has been successfully demonstrated under challenging ecological conditions during a phase two trial [5]. For the past several years, DNA nuclease-based gene-drives have started receiving much emphasis due to the advent of the easily programmable and affordable nuclease CRISPR-Cas9 which has enabled the making of drives with inheritance rate from heterozygous parents close to an astonishing 100%. The mechanism behind the gene-drive technology is that a programmable nuclease is used to cleave a specific, desired site in the genome; the nuclease is then inserted into the site. The gene-drive is configured to remain active in the germline. This in diploid organisms heterozygous for the gene-drive enables the gene-drive to make a double stranded break (DSB) in the homologous chromosome that does not have the gene-drive. Thereafter, the repair of the DSB can take place either by simply end joining (EJ) of the broken strands or by the resection of the DSB, and the synthesis of the intervening strand by using the intact strand as a template, a process called homology direct repair (HDR) [6]. Gene-drive uses HDR, copying the drive from the template strand onto the new intervening strand thereby converting a heterozygote to a homozygote for the drive, a process called Homing (Figure 1). Thus, two factors, the nuclease´s rate of cleavage rate in the germline, and the proclivity of the cells machinery to repair the broken chromosomal segments determine the force of a gene-drive. It is this very phenomenon that allows even small frequencies of gene-drives to spread rapidly throughout a population in just a few generations time even when they carry a fitness cost (Figure 2) [6].

This technology has been recently used to engineer a gene-drive that creates, self-destruct mosquitoes [7]. Female mosquitoes homozygous for the gene-drive exhibited female-male sexual development which rendered them infertile [5]. Further, the drive caused the mosquitoes´ mouths to deform, impairing their ability to bite and transmit the parasite [8]. The gene-drive cascaded throughout the population, rendering the mosquitoes unable to reproduce and within a few generations was able to collapse the entire population. The idea was first tested in 2003 however, it was observed that the gene-drive disappeared after a few generations [7]. This was attributed to the development of resistant alleles, a consequence of EJ. EJ repairs cause the deletions or insertions to occur at the target site which inhibits the nuclease activity. This coupled with the gene-drive's negative fitness cost caused the drive to disappear in the subsequent generations [6]. To counter this problem, a second-generation gene-drive, Ag (QFS)1 which targeted certain sequences in the female specific isoform of the double-sex gene was created. The essential sequences are highly conserved and display high functional constraints rendering them less tolerant to variant alleles [5]. After displaying promising results in small cage studies with non-overlapping generations, Ag (QFS)1 was finally tested in large cages that emulated natural population dynamics. The drive was tested at medium (25%) and low (12.5%) allele frequencies along with the maintenance of a control group. Overlapping generations were maintained through a biweekly introduction of pupae with the startup population. This allowed researchers to assess fecundity, mating success, and other aspects that cannot be measured in single generation studies. Finally, an individual-based stochastic simulation model of the study was created in order to better appreciate the observed population suppression and gene frequency dynamics. Following regulatory guidelines, proper measures were taken to prevent the mosquitoes from escaping the facility [5,8,9].

The gene-drive was able to suppress the entire population within a span of 245 to 311 days. To add to this throughout the study, no resistant mutant alleles capable of coding for functional double sex proteins were identified despite the high selection pressure exerted by Ag (QFS)1 [5]. These positive findings of the study confirm the high efficacy of gene-drive mosquitoes as a self-sustaining, fast acting, and affordable vector control modality under near to natural conditions, forming a bridge between laboratory studies and potential field studies in the future [5-7].

Despite the success of the study, some experts argue that this technology could disrupt the natural balance of things leading to real world problems. Having the potential to alter entire ecosystems, the technology could lead to unprecedented outcomes. In 2017 United states Defense Advanced Research Projects Agency (DARPA) revealed that it was investing US$65 million in programs researching how to halt, counter and reverse gene-drives in the case of any misuse or adverse outcome [9]. Despite these concerns, if properly developed and regulated gene-drives could act as a powerful tool that could help bring an end to the tribulations caused by malaria although it must be stressed that further comprehensive studies must be carried out in a stepwise manner before conducting large scale field trials. Depending on the success of these studies, a limited release of the mosquitoes at field testing sites could be possible within the next 10 years [5,7].

## Conclusion

Malaria remains a major threat to public health, causing high morbidity and mortality across the globe. This also hampers economic activities, causing menace particularly in some of the poorest regions of the world. While traditional methods of malaria control have shown success in curtailing the spread of the disease, the rising number of cases and decreased compliance to preventative measures by the public in recent years warrant superior methods of malaria control. Though still in its developmental stages, Gene-drive technology has the potential to revolutionize malaria control, providing robust, cheap, and self-sustaining means of malaria prevention. The recent success displayed in near to natural conditions is a major development towards possible field trials in the near future. It is imperative to further investigate the drive´s behavior in further near to natural conditions and to address the concerns associated with the technology in order to facilitate the release of the drives into the open.

## References

[1] Tizifa TA, Kabaghe AN, McCann RS, van den Berg H, Van Vugt M, Phiri KS (2018). Prevention Efforts for Malaria. Curr Trop Med Rep.

[2] WHO Malaria.

[3] Buck E, Finnigan NA (2022). Malaria. StatPearls [Internet].

[4] Suh KN, Kain KC, Keystone JS (2004). Malaria. CMAJ.

[5] Hammond A, Pollegioni P, Persampieri T, North A, Minuz R, Trusso A (2021). Gene-drive suppression of mosquito populations in large cages as a bridge between lab and field. Nat Commun.

[6] Hammond AM, Kyrou K, Bruttini M, North A, Galizi R, Karlsson X (2017). The creation and selection of mutations resistant to a gene-drive over multiple generations in the malaria mosquito. PLoS Genet.

[7] Geddes L (2021). Genetic engineering test with mosquitoes ‘may be game changer’ in eliminating malaria. The Guardian.

[8] Stein R (2021). How An Altered Strand Of DNA Can Cause Malaria-Spreading Mosquitoes To Self-Destruct.

[9] Scudellari M (2019). Self-destructing mosquitoes and sterilized rodents: the promise of gene-drives. Nature.

